# Exploration of immune-related genes in high and low tumor mutation burden groups of chromophobe renal cell carcinoma

**DOI:** 10.1042/BSR20201491

**Published:** 2020-07-23

**Authors:** Lei Li, Xi Chen, Lu Hao, Qiuyan Chen, Haosheng Liu, Qing Zhou

**Affiliations:** 1 Central Laboratory, People’s Hospital of Baoan District, Shenzhen, China; 2 Science and Education Department, Shenzhen Baoan Shiyan People’s Hospital, Shenzhen, China; 3 School of Biological Science and Medical Engineering, Beihang University, Beijing, China

**Keywords:** chromophobe renal cell carcinoma, immunotherapy, TCGA, tumor mutation burden

## Abstract

Renal cell carcinoma (RCC) is one of most common cancers with gradually increasing incidence and high mortality. Chromogenic RCC (chRCC) is the third most common histological subtype of RCC, accounting for approximately 5–7% of RCC. In our study, the transcriptome expression profile data (*n*=89) of chRCC, corresponding clinical data (*n*=113) and the somatic mutation data (*n*=66) were obtained from the TCGA database. We first analyzed the mutation data of chRCC patients and divided chRCC patients into high and low tumor mutation burden (TMB) groups based on the median TMB. We found that high TMB was significantly associated with worse prognosis and could promote tumor metastasis and development. Moreover, four different immune-related genes (BIRC5, PDGFRL, INHBE, IL20RB) were also identified. We found that BIRC5 was significantly overexpressed in the high TMB group and correlated with worse prognosis. The results of univariate and multivariate COX analyses demonstrated that BIRC5 (hazard ratio (HR) = 2.094) may serve as a prognostic indicator for patients with chRCC with high TMB. In addition, we identified the possible functional pathways of BIRC5 through gene set enrichment analysis (GSEA) enrichment. A positive correlation was obtained between BIRC5 and the abundance of CD4^+^ T cells. The results of our study revealed their correlation between the immune-related genes and clinicopathologic features as well as potential functional pathways as well as immune infiltrating cells, which may provide more data about the development of chRCC immunotherapy.

## Introduction

Kidney cancer is well known as the third most common malignant tumor in the urinary system after prostate cancer and bladder cancer [1–3]. According to the literature, the globally estimated new cases and deaths of kidney cancer were 403262 and 175098 in 2018, respectively [4]. The prognosis of RCC is poor, with the overall survival of stage IV RCC patients to be 10–15 months. Approximately 90% of kidney cancers are renal cell carcinoma (RCC), including clear cell RCC (ccRCC), chromogenic RCC (chRCC) and papillary RCC (pRCC). chRCC is the third most common histological subtype of RCC, accounting for approximately 5–7% of RCC [5–7]. There are many treatments for chRCC, but immunotherapy is considered to be the most promising and immune checkpoint inhibitor proven useful for RCC [8,9]. However, the role of immunotherapy in chRCC is far from fully clarified.

Increasing evidences revealed that immunotherapy is the most effective way to treat advanced or aggressive cancer [10–13]. The blockade of these immune checkpoints has translated into effective strategies for cancer immunotherapy. Immune checkpoints have achieved great success in suppressing lung cancer, breast cancer and melanoma [14–17]. Tumor mutation burden (TMB) is referred to the number of mutations that exist within a megabase of genomic territory [18]. Previous studies suggest that the TMB is closely related to the efficacy of immunotherapy in most cancer types [19–22]. Moreover, high TMB predicts immunotherapy benefit and TMB can predict survival after immunotherapy across multiple cancer types [20,23]. However, only approximately 20% of cancer patients can benefit from immunotherapy [24]. What is worse is that few related studies have focused on primary chRCC in the TMB subgroup. These sobering data illustrate a critical need to determine the immunotherapy response mechanism for primary chRCC.

In the present study, we analyzed significantly different immune-related genes in the high and low TMB subgroups, explored the prognostic role of immune-related genes in chRCC and its potential correlation with immune-infiltrating cells. The results of our study may provide sufficient information for patient prognosis prediction and additional choice for the immunotherapy of primary chRCC.

## Materials and methods

### Datasets

The primary chRCC transcriptome expression profile (*n*=89) and corresponding clinical data (*n*=113) including age, gender, tumor grade, pathological stage, etc. were downloaded from the TCGA database (https://tcga-data.nci.nih.gov/tcga/). Somatic mutation data (*n*=66) were also obtained from the TCGA database with data type of ‘Masked Somatic Mutation’. Meanwhile, we obtained a list of the immune-related genes from the ImmPort database (https://www.immport.org/) [25].

### Calculation of TMB scores and prognostic analysis

TMB is defined as the total number of mutations per megabase in tumor tissue. ‘maftools’ R package was used to calculate the mutations of each sample in chRCC, and all the chRCC samples were divided into low and high TMB groups based on median data. It was combined with clinical information to analyze the relationship between TMB and Stage and tumor metastasis. In addition, we used the ‘survival’ R package to analyze the relationship between high/low TMB groups and prognosis.

### Identification of differentially expressed genes and immune-related genes

We used the ‘Limma’ R package to identify differentially expressed genes in the high and low TMB groups with a fold change (FC) of 2 and *P*-value of 0.05. And the result was visualized with the ‘pheatmap’ package. Compared with a list of immune-related genes from the immunology database (Immport), we determine the immune-related genes from all differentially expressed genes.

### Functional enrichment analysis

In order to identify potential functions and approaches of differentially expressed genes, enrichment analysis including GO (Gene Ontology) function and KEGG (Kyoto Encyclopedia of Genes and Genomes) pathway enrichment analyses were performed using ‘clusterProfiler’ R package.

### Analysis of immune-related genes in high and low TMB groups

The immune-related genes were selected to further evaluate the prognostic value of differential immune-related genes in patients with low and high TMB levels. In addition, we compared the expression of four immune-related genes in the high and low TMB groups through the ‘beeswarm’ package, and then further evaluated their prognostic value using univariate and multivariate factor Cox regress analyses.

### Gene Set Enrichment Analysis

We performed a Gene Set Enrichment Analysis (GSEA) analysis using the GSEA_4.0.3 software in order to further understand the BIRC5-related pathways. We divided the patients in the TCGA cohort into two groups based on TMB score for GSEA of BIRC5 and selected the c2.cp.kegg.v7.0.symbols.gmt gene set as the reference gene set, with a nominal *P*-value of <5% as a standard [26].

### Statistical analysis

All analyses were performed using R software (version 3.6.2). The Cox regression analysis was performed based on the ‘survival’ R package. The ‘Limma’ package was mainly used for the analysis of differences. A *P*-value <0.05 was considered significant.

## Results

### The landscape of mutation profiles in chRCC

**Figure 1:**
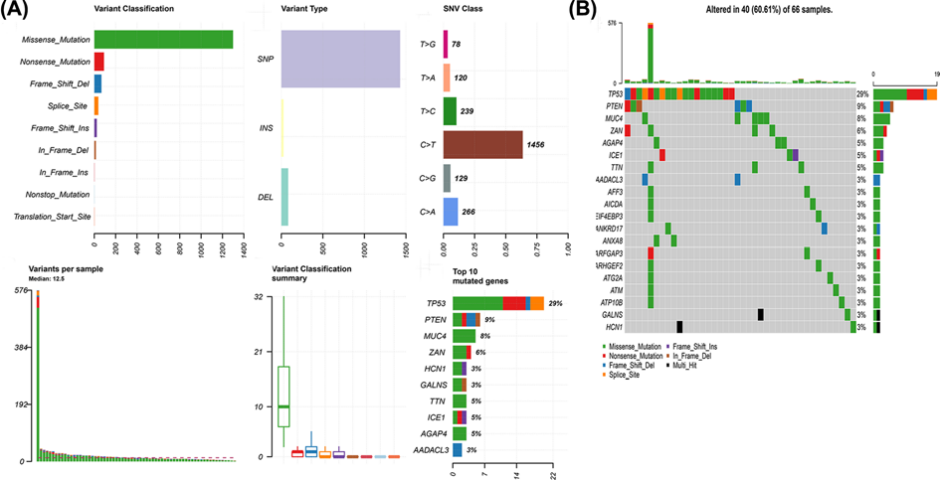
TCGA chRCC mutation cohort (**A**) Overview of TCGA chRCC cohort mutations. (**B**) Waterfall of the top 20 mutated genes in the TCGA chRCC cohort.

Obtained from the TCGA database, somatic mutation data (*n*=66) were analyzed with R, and the results were visualized with ‘maftools’ package. We further classified these mutations according to different categories. As shown in Figure 1A, missense mutation was the most common type of variant classification, and single nucleotide polymorphisms occurred more frequently than insertions or deletions. C > T was the most common single-nucleotide variation (SNV) in chRCC (Figure 1A). Then we calculated the number of base changes in each sample, with different colors representing different mutation types (Figure 1A). The waterfall chart revealed the top 20 mutant genes of the mutation profile in chRCC, including TP53 (29%), PTEN (9%), MUC4 (8%), ZAN (6%), HCN1 (3%), TTN (5%), ICE1 (5%), AGAP4 (5%), and AADACL3 (3%) et al. (Figure 1B).

### Correlation of TMB with prognosis, clinicopathological characteristics, and tumor grades of chRCC patients, and functional enrichment analysis

**Figure 2:**
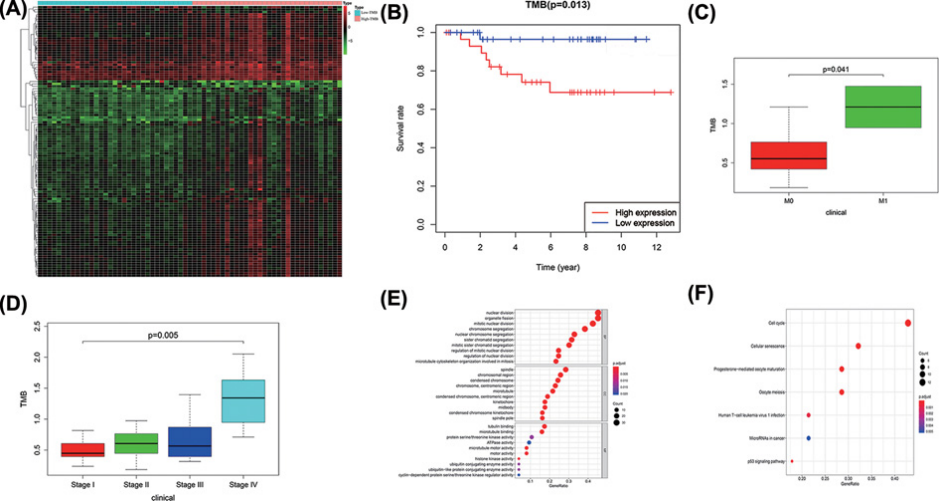
TMB correlation analysis (**A**) Heatmap of differentially expressed genes in high and low TMB groups. (**B**) Kaplan–Meier survival analysis. (**C**) Relationship between TMB and tumor metastasis. (**D**) Relationship between TMB and tumor stage. (**E,F**) GO and KEGG results.

Based on the median TMB, we divided chRCC into high TMB group (*n*=32) and low TMB group (*n*=33), and analyzed its gene expression profile to identify DEGs with a false discovery rate (FDR) of 0.05 and an FC of 1.5, and the result was visualized with a heatmap (Figure 2A). Kaplan–Meier survival analysis revealed that patients with high TMB were associated with worse prognosis (*P*=0.013, Figure 2B). Next, we analyzed the relationship between TMB and tumor metastasis, and the results showed that high TMB may promote tumor metastasis (*P*=0.041, Figure 2C). And TMB levels correlate with advanced tumor grade (*P*=0.005, Figure 2D). Finally, we used the ‘clusterProfiler’ R package to explore the potential functions and pathways of these genes. A total of 291 GO terms and 7 pathways were identified (*P*<0.05, enrichment score of >1.5). The results showed that the top cancer-related biological processes were associated with the regulation of mitotic nuclear division, nuclear division, and organelle fission (Figure 2E), and the results suggested that these genes were mainly enriched in the cancer‐related signaling pathway, such as cell cycle, progesterone-mediated oocyte maturation, cellular senescence, oocyte meiosis, et al. (Figure 2F).

### Identification of immune-related genes

**Figure 3:**
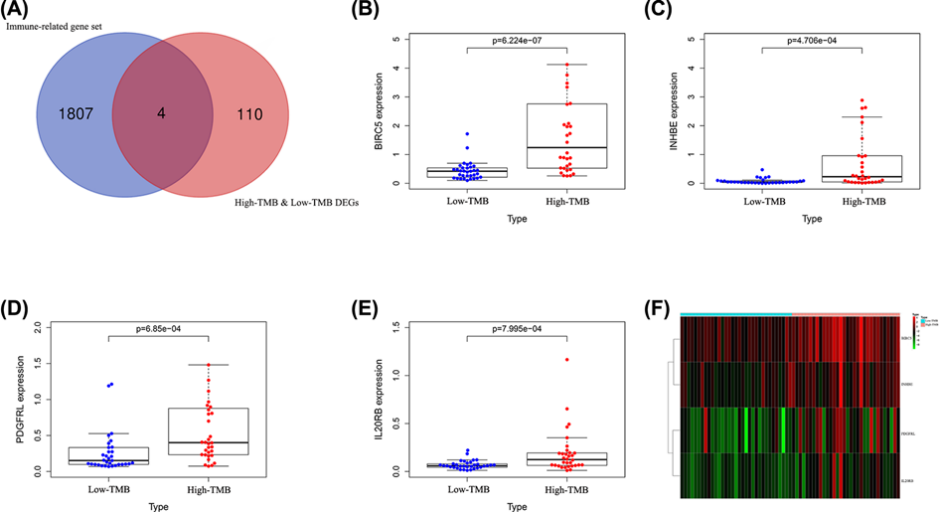
Identification of immune-related genes in high and low TMB groups (**A**) Four immune-related genes. (**B–E**) Expression levels of four immune-related genes in high and low TMB groups. (**F**) Heatmap of differences in four immune-related genes.

A total of 114 DEGs were identified by comparing the high and low TMB groups (Supplementary Table S1). We also downloaded a list of immune-related genes from the immunology database (Immport), from which we identified four immune-related genes, including BIRC5, PDGFRL, INHBE, and IL20RB (Figure 3A).

### Expression levels of four immune-related genes in high and low TMB groups

We extracted the expression data of four immune-related genes from the expression file, then compared their expression in the high and low TMB groups, and visualized them through the ‘beeswarm’ package. From the results, we found that BIRC5, PDGFRL, INHBE, and IL20RB were highly expressed in the high TMB group (Figure 3B–E). The heatmap in Figure 3F showed the expression of four immune-related genes in the high and low TMB groups.

### The correlation between four immune-related genes with TMB, tumor metastasis, patients’ survival, and immune checkpoint genes

**Figure 4:**
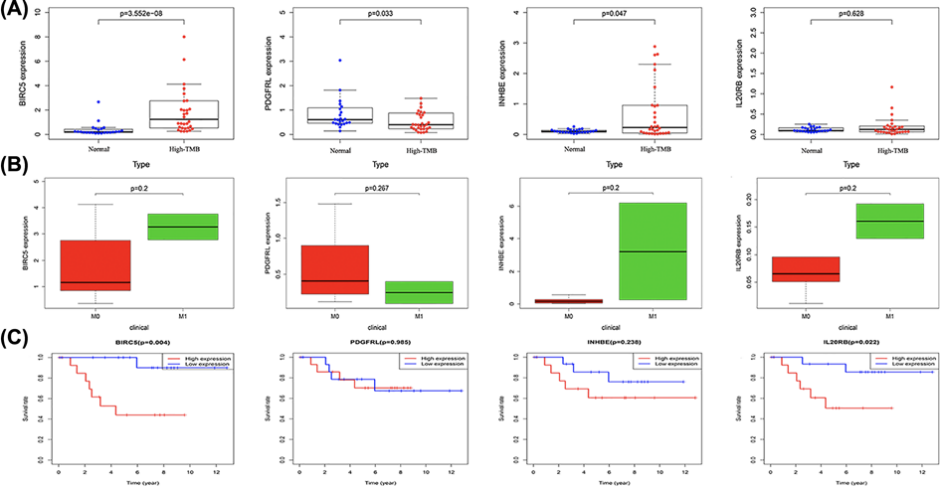
Correlation of four immune-related genes with high TMB group (**A**) Expression levels of four immune-related genes in normal group and high TMB groups. (**B**) Expression levels of four immune-related genes in high TMB group patients with and without tumor metastasis. (**C**) The overall survival of high TMB group patients with high and low expression levels of four immune-related genes.

**Figure 5:**
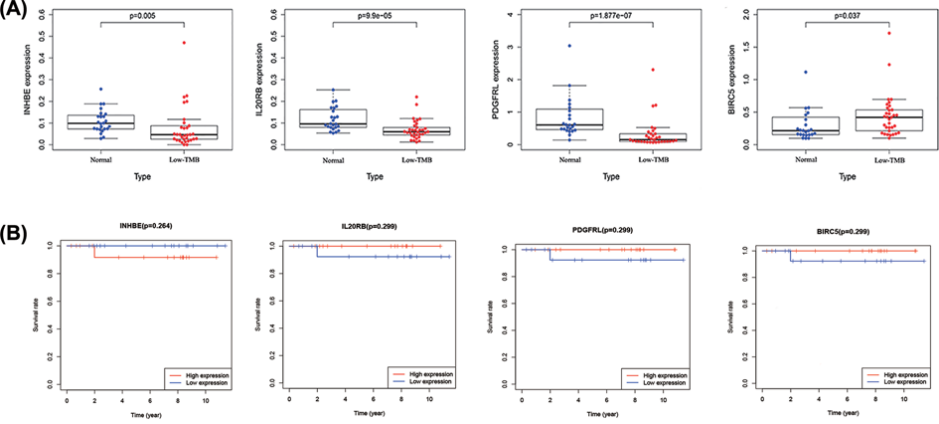
Correlation of four immune-related genes with low TMB group (**A**) Expression levels of four immune-related genes in normal group and low TMB groups. (**B**) The overall survival of low TMB group patients with high and low expression levels of four immune-related genes.

In order to better understand the role of these four immune-related genes in chRCC, we divided chRCC patients into high and low TMB groups, and analyzed the expression levels of these four immune-related genes in high and low TMB groups, and then analyzed their correlation with tumor metastasis and patients’ prognosis. In the results of the high TMB group (Figure 4A), we found that BIRC5 (*P*=3.552e-08) and INHBE (*P*=0.047) were significantly up-regulated in the high TMB group, while PDGFRL (*P*=0.033) was significantly down-regulated in the high TMB group. However, all these four immune-related genes were not related to tumor metastasis (Figure 4B). We then found that high TMB chRCC patients with BIRC5 (*P*=0.004) and IL20RB (*P*=0.022) were correlated with poor prognosis (Figure 4C). As a result in low TMB chRCC group, we found that INHBE (*P*=0.005), IL20RB (*P*=9.9e-05), and PDGFRL (*P*=1.877e-07) were down-regulated in the low TMB group (Figure 5A). In contrast, BIRC5 (*P*=0.037) was up-regulated in the low TMB group (Figure 5A). Moreover, we revealed that the expression of these four immune-related genes would not affect the prognosis of chRCC patients with low TMB (Figure 5B). The correlation between the expression of four immune-related genes and the reported immune checkpoint genes were also analyzed, which revealed that PDGFRL was certainly positively correlated with the immune checkpoint genes (Supplementary Table S2).

### Prognosis of BIRC5 in high TMB group

**Figure 6:**
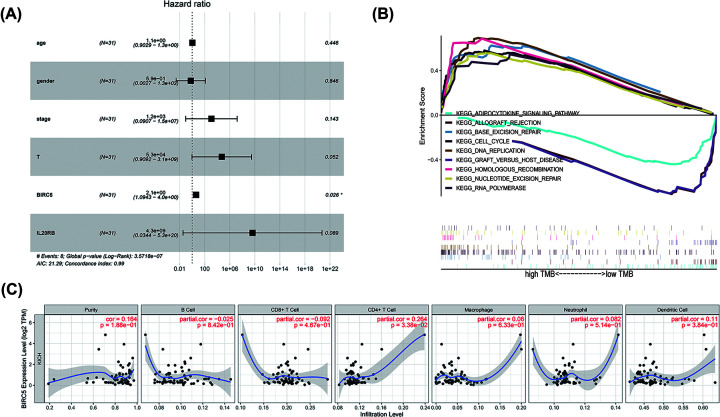
Cox analysis, GSEA, and tumor-infiltrating immune cells analysis of BIRC5 (**A**) Cox analysis of BIRC5. (**B**) GSEA of BIRC5 based on based on TMB score. (**C**) Tumor-infiltrating immune cells analysis of BIRC5.

Previous results indicated that BIRC5 and IL20RB were significantly associated with the prognosis of patients with high TMB. In order to further study the prognosis in the chRCC high TMB group, we further performed a COX regression analysis in the high TMB group (Table 1). First, we found that stage (hazard ratio (HR) = 9.985, 95% confidence interval (CI) = 2.481–40.187, *P*=0.001), T (HR = 19.829, 95% CI = 2.487–158.045, *P*=0.005), and BIRC5 (HR = 1.177, 95% CI = 1.062–1.303, *P*=0.002) were significantly correlated with prognosis in univariate COX analysis. After that, we performed a multivariate COX analysis, which revealed that BIRC5 (HR = 2.094; 95% CI = 1.094–4.010; *P*=0.026) was independently associated with a worse prognosis (Figure 6A, Table 1).

**Table 1:** Univariate and multivariate Cox regression analyses

Parameter	Univariate Cox analysis			Multivariate Cox analysis		
	HR	95% CI	P	HR	95% CI	P
Age	1.040	0.970–1.116076	0.262	1.067	0.970–1.261	0.445
Gender	2.428	0.488–12.0696	0.277	0.587	0.488–125.377	0.845
Stage	9.985	2.481–40.187	0.001	1175.509	2.481–15228757	0.143
T	19.829	2.487–158.045	0.005	52662.11	2.487–3.05E+09	0.052
BIRC5	1.177	1.062–1.303	0.002	2.094934	1.062–4.010	0.025
IL20RB	4.66	0.603–36.015	0.140	4.27E+09	0.603–5.30E+20	0.088

*P*<0.05 has statistical significance.

### GSEA and immune infiltration of BIRC5

GSEA was performed to explored the BIRC5 associated-functions in chRCC. The results revealed prominent enrichment of signatures related in the base excision repair, cell cycle, DNA replication, homologous reorganization, nucleotide excision repair, and RNA polymerase in the high TMB group (Figure 6B, Table 2). In addition, adipocytokine signaling pathway, allograft rejection, and graft versus host disease were enriched in the low TMB group group (Figure 6B, Table 2).

**Table 2:** GSEA

Name	ES	NES	NOM *P*-val	FDR q-val
KEGG_ADIPOCYTOKINE_SIGNALING_PATHWAY	−0.45	−1.64	0.017	0.323
KEGG_ALLOGRAFT_REJECTION	−0.72	−1.68	0.031	0.481
KEGG_BASE_EXCISION_REPAIR	0.62	1.75	0.014	0.765
KEGG_CELL_CYCLE	0.58	1.74	0.010	0.283
KEGG_DNA_REPLICATION	0.69	1.60	0.024	0.331
KEGG_GRAFT_VERSUS_HOST_DISEASE	−0.72	−1.65	0.036	0.421
KEGG_HOMOLOGOUS_RECOMBINATION	0.69	1.73	0.014	0.233
KEGG_NUCLEOTIDE_EXCISION_REPAIR	0.55	1.60	0.041	0.375
KEGG_RNA_POLYMERASE	0.55	1.68	0.017	0.288

NOM *P*-val <0.05 has statistical significance.

To further analyze the function of BIRC5 in the high TMB group, we used TIMER to verify the correlation between BIRC5 and immune cell infiltration levels (Figure 6C). We found a positive correlation between BIRC5 expression and the abundance of CD4 ^+^ T cells (Cor = 0.264; *P*=3.38e-02).

## Discussion

At present, the immunotherapy for RCC is mainly based on immune checkpoints of PD-1 and PD-L1 inhibitors [27–29]. However, the immunotherapy of chRCC is still insufficient. TMB has proven to be a determinant of immune-related survival in many tumor patients and is extremely important in the treatment of tumors [30–33]. TMB can also be used as one of the indicators for the treatment of RCC patients, but the current TMB research on chRCC patients is still insufficient [34–36]. The prognostic role of immune-related genes in high and low TMB groups and their relevance to immunotherapy have not yet been explored. Thus, this research investigated the prognostic role of immune-related genes and the potential association with immune infiltrate cells in chRCC.

In the current study, chRCC somatic mutation data were obtained from the TCGA database and then divided into high TMB and low TMB groups based on the median. We then analyzed the immune gene differences in the high and low TMB groups and analyzed the correlation of immune-related gene function and prognosis. As a result, it was found through research that mutations in chRCC are also common, and their mutations are mainly missense mutations. Moreover, SNP was the most common variant type. Actually, somatic missense mutations strongly contribute to the generation of novel tumor epitopes [37] Previous studies have demonstrated the significance of missense mutation and SNP in tumorigenesis, progression, and prognosis in various cancer types, including bladder cancer [38–41]. The three most frequently mutated genes were TP53, PTEN, and MUC4. TP53 is one of the famous tumor suppressor genes reported to regulate the cell cycle thus inhibits the development of cancerous cells [42]. P53 protein maintains genome stability and prevents the occurrence of genomic mutation [43]. PTEN was referred as a dormant tumor suppressor in RCC and associated with associated with patients’ prognosis [44].

We divided chRCC patients into high and low TMB groups based on TMB levels and analyzed the differences between high and low TMB groups. We found that there was a significant difference between high TMB and low TMB groups. High TMB was correlated with a worse prognosis and would promote tumor metastasis and development. In fact, another study about ccRCC also suggested that patients with higher TMB tended to be with a worse prognosis [45]. Moreover, Chuanjie et al. found that ccRCC with higher TMB levels are associated with higher tumor grades and advanced pathological stages [46].

Another important finding of our study was that four differentially expressed immune-related genes (BIRC5, INHBE, PDGFRL, and IL20RB) were identified in the high and low TMB groups. BIRC5 and IL20RB were correlated with the worse prognosis in high TMB groups analysis. Previous studies revealed that BIRC5 could promote tumor cell proliferation in RCC [46]. Moreover, BIRC5 was found to be a biomarker for prognosis and therapy in other types of cancers, including lung cancer and pancreatic cancer [47,48]. Interestingly, IL20RB was also suggested as a prognosis biomarker in pRCC, and promoted cell proliferation, invasion and migration [49]. Our study further highlighted the significance of BIRC5 and IL20RB in the tumorigenesis and progress of RCC.

BIRC5, selected for further study and univariate as well as multivariate Cox analyses, demonstrated that it can be used as an independent prognostic factor for chRCC. Moreover, the result of GSEA revealed that BIRC5 was involved in base excision repair, cell cycle, DNA replication, homologous reorganization, nucleotide excision repair, RNA polymerase, and adipocytokine signaling pathway in chRCC. DNA replication is one of the fundamental biological processes in which dysregulation can cause genome instability [50]. And DNA replication errors are the main drivers of cancers, including RCC [51]. The normal process of cell division occurs via the cell cycle, and dysregulation of cell cycle would result in sustained unscheduled cell growth, proliferation, a hallmark of cancer [52]. In hepatocellular carcinoma, down-regulation of BIRC5 could induce cancer cell apoptosis and cell cycle arrest [53]. Thus, BIRC5 may regulate RCC development via cell cycle.

In our study, we also found that BIRC5 in chRCC was involved in immune cell infiltration. Actually, another study also found that BIRC5 was associated with immune cell infiltration and served as a prognostic biomarker and therapeutic target for HCC [54]. In chRCC, BIRC5 showed positive correlation with CD4^+^ T cells. Previous study revealed that CD4^+^ T cells could promote tumor cell proliferation in RCC [55]. Moreover, another study found that CD4^+^ T cells in RCC patients were associated with favorable prognosis [56]. Therefore, BIRC5 may also regulate tumor cell biological process, thus affecting the prognosis of chRCC via CD4^+^ T cells. And further studies should test this hypothesis.

Admittedly, our research also had some limitations. First of all, the results of the current study were not validated using another independent patient cohort. Moreover, it would be better if *in vitro* or *in vivo* experiments were performed to validate our findings.

In summary, our study shows that high TMB in chRCC patients correlated with worse prognosis, while BIRC5 in the high TMB group can be used as an independent prognostic indicator of chRCC. We also predict that BIRC5-enriched functional pathways and potentially affected immune-infiltrating cells, and this information may contribute to the development of chRCC therapy.

## Supplementary Material

Supplementary Tables S1-S2

## Abbreviations

ccRCC: clear cell renal cell carcinoma
chRCC: chromogenic renal cell carcinoma
CI: confidence interval
DEG: differentially expressed genes
FC: fold change
GO: gene ontology
GSEA: gene set enrichment analysis
HR: hazard ratio
PD-L1: programmed death ligand 1
PD-1: programmed cell death protein 1
pRCC: papillary renal cell carcinoma
RCC: renal cell carcinoma
SNV: single-nucleotide variation
TCGA: the cancer genome atlas
TIMER: tumor immune estimation resource
TMB: tumor mutation burden
