# Supplementary material for: Exploration of immune-related genes in high and low tumor mutation burden groups of chromophobe renal cell carcinoma
Source: Biosci Rep. 2020 Jul 23;40(7):BSR20201491. doi: 10.1042/BSR20201491 (PMC7378265; doi:10.1042/BSR20201491)
Supplement: Supplementary Tables S1-S2 [file BSR-2020-1491_supp.pdf]

Supplementary Table 1. DEGs were identified by comparing the high and low TMB groups.

gene	conMean	treatMean	logFC	pValue	fdr
BUB1B	0.412921	1.438073	1.800198	5.91E-06	0.003079
DEPDC1	0.07115	0.55248	2.95698	2.90E-05	0.0061
ESPL1	0.154083	0.602601	1.967497	0.000403	0.028437
SGO1	0.09083	0.417472	2.200441	2.38E-05	0.00578
GTSE1	0.330921	1.520785	2.200256	4.56E-05	0.007129
TEX22	0.295812	0.612507	1.050045	2.71E-05	0.0061
PRPH	0.060289	0.405759	2.750662	7.53E-05	0.009807
IQGAP3	0.158893	0.74652	2.23212	1.29E-05	0.004151
OIP5	0.381052	0.944089	1.308933	5.91E-06	0.003079
CDHR2	0.184838	0.387872	1.069318	0.000122	0.012611
KIF2C	0.50051	1.77301	1.82473	0.000527	0.033904
TOP2A	0.694169	3.510626	2.338369	0.000138	0.013805
SPAG5	0.378773	1.413945	1.900321	6.65E-05	0.009099
NDC80	0.33155	1.173204	1.823155	0.000586	0.035988
CAMK2N2	0.051999	0.872207	4.068123	4.11E-05	0.006987
C17orf53	0.201899	0.558489	1.467895	3.80E-06	0.002445
MND1	0.742042	1.503842	1.01908	0.000556	0.034926
ASF1B	1.303345	4.265269	1.710418	1.12E-05	0.003962
TONSL	0.579797	1.222993	1.076797	4.86E-05	0.007285
CENPF	0.312107	1.618519	2.374561	1.05E-05	0.003949
NUSAP1	1.116088	4.083218	1.871256	1.82E-05	0.00497
CDC20	0.782562	4.986412	2.671725	5.50E-06	0.003079
CDCA5	0.654679	2.071007	1.661473	5.18E-05	0.007656
KIF4A	0.379717	1.668915	2.135914	1.94E-05	0.005065
CDCA2	0.148688	0.738638	2.312584	1.70E-05	0.004888
NEK2	0.130077	0.841057	2.692834	6.83E-06	0.003249
PCLAF	0.412138	1.450899	1.815748	0.000259	0.020686
POC1A	1.199222	3.055887	1.349493	1.38E-05	0.004201
CENPE	0.165869	0.581098	1.808735	0.000122	0.012611
SULT1C3	1.632363	0.042709	-5.25628	0.000942	0.048844
RRM2	0.288366	1.478689	2.358344	0.000184	0.016243
PIF1	0.079638	0.429751	2.431968	1.12E-05	0.003962
RBM11	0.334279	0.141411	-1.24116	0.000197	0.017008
CDK1	0.69591	2.122753	1.608964	0.000155	0.015245
SLC16A1	3.931963	8.11177	1.044767	1.38E-05	0.004201
TRIP13	0.301378	1.088584	1.852804	0.000164	0.015331
ZMYND10	0.103133	0.312395	1.598863	0.000403	0.028437
KIFC1	0.423297	1.604954	1.92279	2.54E-05	0.005914
CENPW	1.448968	4.104598	1.502215	2.90E-05	0.0061
CDCA3	0.212035	0.815275	1.942988	1.10E-06	0.001205
CCNA2	0.971127	2.822215	1.539096	3.76E-05	0.006977
UBE2T	1.545001	3.95731	1.356913	4.28E-05	0.006987
EIF4EBP1	5.876176	18.38878	1.645877	0.00076	0.042608
ASPM	0.091522	0.571534	2.642653	3.10E-05	0.006391
KIF23	0.409393	1.390652	1.764202	8.01E-05	0.010069
NEIL3	0.065142	0.376853	2.532352	9.13E-08	0.000333
EZH2	0.564379	1.550608	1.458097	3.80E-06	0.002445
CCNB2	0.675592	2.923355	2.1134	9.13E-08	0.000333
PLK1	0.310275	1.748328	2.494355	2.91E-07	0.000636
PBK	0.56722	3.163265	2.479435	4.75E-06	0.002884
TROAP	0.252344	1.335396	2.403807	6.22E-07	0.000973
ANKRD65	0.73319	0.364449	-1.00847	0.000231	0.019035
TPX2	1.063401	5.654511	2.410716	3.27E-06	0.002445
FAM83D	0.323762	1.666389	2.36372	3.53E-06	0.002445

UBE2S	3.486671	7.164141	1.038944	6.83E-06	0.003249
APLP1	0.527874	1.609326	1.60819	9.61E-05	0.011187
INHBE	0.244946	0.837988	1.774464	0.000471	0.0314
CDC25A	0.446598	1.018365	1.189205	9.05E-05	0.010876
CENPI	0.108856	0.42362	1.960348	8.01E-05	0.010069
ANLN	0.367973	1.939302	2.397867	3.31E-05	0.006574
TMEM63C	0.475727	1.294408	1.444086	0.000931	0.048514
CDT1	1.655898	3.551219	1.100701	0.000841	0.045567
IL20RB	0.069403	0.473551	2.770447	0.00076	0.042608
CLGN	3.30024	10.37815	1.652907	0.000425	0.029636
WDR62	0.143968	0.460008	1.675913	7.97E-07	0.001089
STMN1	4.703173	10.37037	1.140761	8.47E-06	0.003563
UHRF1	0.228155	0.906994	1.99108	7.08E-05	0.009331
CDKN2C	1.508074	3.488628	1.209953	0.000885	0.046786
NCAPG	0.256965	1.158674	2.172832	0.000231	0.019035
DLGAP5	0.21362	1.309904	2.616344	3.03E-06	0.002445
CDC25C	0.083604	0.446398	2.416694	0.000164	0.015331
KIF18B	0.11862	0.739883	2.640946	3.53E-05	0.006769
ARHGAP11A	0.422766	1.791198	2.082994	2.44E-07	0.000636
MKI67	0.368258	1.653794	2.166992	1.59E-05	0.004689
QRFPR	0.56936	0.077001	-2.88639	0.000161	0.015331
RAD54L	0.166649	0.509424	1.612051	2.90E-05	0.0061
BIRC5	0.440444	2.632936	2.579642	6.22E-07	0.000973
KIF15	0.137182	0.566535	2.046071	1.29E-05	0.004151
FOXM1	0.832083	3.589322	2.108911	9.13E-08	0.000333
STEAP1	0.249004	1.381779	2.472284	0.000349	0.025995
SPC24	0.360068	1.517589	2.075439	4.28E-05	0.006987
KIF20A	0.351274	1.880356	2.420338	2.08E-05	0.005291
STEAP4	1.508639	0.614646	-1.29542	0.000259	0.020686
E2F2	0.083671	0.339212	2.019398	0.000184	0.016243
MYBL2	0.35429	2.719174	2.940166	0.000164	0.015331
MANEAL	1.614257	4.010293	1.312837	6.65E-05	0.009099
UBE2C	4.296065	13.31937	1.632438	0.000259	0.020686
BUB1	0.41591	1.531524	1.880624	5.87E-05	0.008345
HJURP	0.127563	0.642495	2.332479	1.05E-05	0.003949
PRC1	1.270378	3.74299	1.558933	7.89E-06	0.003451
TEDC2	0.349657	0.931853	1.414163	1.05E-05	0.003949
CEMIP	0.256387	5.559248	4.438498	0.000685	0.040284
CDKN3	0.570865	3.000444	2.393955	2.81E-06	0.002445
CEP55	0.271945	1.942988	2.83689	5.87E-05	0.008345
NUF2	0.137878	0.662182	2.26384	7.34E-06	0.003346
DSCC1	0.261019	0.725223	1.47427	0.000617	0.037299
BEST1	0.358889	0.98518	1.456851	0.000102	0.011636
NCAPH	0.255199	0.8503	1.736351	0.000556	0.034926
MELK	0.328605	1.409314	2.100566	1.29E-05	0.004151
PTTG1	0.970803	5.240227	2.432378	2.22E-05	0.00553
DNASE1L3	4.049073	1.560327	-1.37574	0.000184	0.016243
CDC6	0.19329	0.5682	1.55563	0.00076	0.042608
CDCA8	0.529942	2.222533	2.068298	3.31E-05	0.006574
AURKB	0.221503	1.636721	2.885411	2.54E-05	0.005914
SKA3	0.128803	0.510251	1.98604	0.000184	0.016243
CENPA	0.158955	1.072694	2.754551	1.10E-06	0.001205
KIF11	0.394239	1.153201	1.548501	4.86E-05	0.007285
PKMYT1	0.201565	0.817447	2.01988	2.81E-06	0.002445
PDGFRL	0.317206	1.4585	2.200993	0.000685	0.040284
PSRC1	0.242555	0.68337	1.494357	0.000342	0.025628
CCNB1	2.611652	8.502456	1.702917	7.08E-05	0.009331

E2F1	3.704407	8.588228	1.213118	5.52E-05	0.008046
SKA1	0.152827	0.708747	2.213372	9.05E-05	0.010876

Supplementary Table2. Correlation analysis between immune-related genes and the reported immune checkpoint genes (ICGs) inchRCC.

**ICGs**	BIRC5		PDGFRL		INHBE		IL20RB	
	Cor	P-value	Cor	P-value	Cor	P-value	Cor	P-value
CD8A	-0.047	0.708	0.291	*	0.035	0.778	0.122	0.33
CD8B	-0.139	0.265	0.254	*	0.058	0.644	0.169	0.174
CD3D	-0.033	0.79	0.244	*	0.066	0.6	0.168	0.177
CD3E	0.001	0.992	0.289	*	0.117	0.349	0.197	0.113
CD2	0.01	0.934	0.292	*	0.08	0.522	0.178	0.153
CD19	0.015	0.905	0.236	0.0559	0.003	0.983	0.077	0.536
CD79A	-0.021	0.866	0.235	0.0572	0.056	0.655	0.214	0.085
CD86	-0.074	0.552	0.261	*	0.088	0.48	0.122	0.327
CSF1R	-0.094	0.453	0.219	0.077	0.071	0.573	0.103	0.408
CCL2	-0.106	0.397	0.245	*	-0.018	0.883	0.13	0.297
CD68	-0.03	0.812	0.291	*	0.047	0.71	0.134	0.283
IL10	-0.166	0.182	0.254	*	-0.076	0.544	0.06	0.633
NOS2	-0.115	0.356	0.145	0.245	-0.191	0.124	-0.085	0.495
IRF5	-0.006	0.962	0.166	0.184	0.116	0.353	0.074	0.556
PTGS2	-0.021	0.867	0.087	0.489	-0.009	0.94	0.08	0.523
CD163	0.095	0.449	0.332	**	0.024	0.848	0.169	0.174
VSIG4	0.185	0.137	0.414	***	0.126	0.313	0.262	*
MS4A4A	-0.082	0.51	0.247	*	0	0.999	0.119	0.341
CEACAM8	0.103	0.408	0.091	0.467	-0.071	0.569	0.003	0.982
ITGAM	-0.121	0.333	0.198	0.111	0.03	0.813	0.075	0.547
CCR7	-0.069	0.584	0.247	*	0.086	0.493	0.135	0.278
PDL1	0.118	0.346	0.273	*	0.135	0.278	0.217	0.0808
CTLA4	0.122	0.33	0.364	**	0.199	0.109	0.193	0.121
LAG3	0.051	0.686	0.286	*	0.344	**	0.256	*
HAVCR2	-0.181	0.147	0.097	0.436	-0.002	0.988	-0.036	0.776
GZMB	-0.049	0.694	0.19	0.125	0.115	0.359	0.079	0.528
PDCD1	-0.037	0.768	0.245	*	0.121	0.334	0.086	0.494

*P<0.05,**P<0.01,***P<0.001
